# Foreword to the focus issue: advancements of functional materials with nanoarchitectonics as post-nanotechnology concept in materials science

**DOI:** 10.1080/14686996.2023.2205327

**Published:** 2023-04-25

**Authors:** Katsuhiko Ariga

**Affiliations:** a International Center for Materials Nanoarchitectonics (MANA), National Institute for Materials Science (NIMS), Tsukuba, Japan; b Department of Advanced Materials Science, Graduate School of Frontier Sciences, The University of Tokyo, Kashiwa, Chiba, Japan

Human life depends on the materials available at a given time. Therefore, science and technology of advanced materials is the key to human development. In particular, advances in the fields of material fabrication and functional elucidation that have emerged over the past century have brought about rapid development of human society. The creation and rapid development of organic chemistry, inorganic chemistry, polymer chemistry, coordination chemistry, supramolecular chemistry, biochemistry, and other material sciences have brought about not only progress in academic fields but also the development of human society itself. In addition to these materials divisions, the latter half of the 20th century saw the inception of the revolutionary concept of nanotechnology. Nanotechnology is a game changer that has revolutionized the course of scientific research. Direct observation, functional evaluation, and manipulation at the atomic, molecular, and nano-level became possible with the development of nanotechnology. Nanotechnology innovations have unlocked questions at the nanoscale. It has become clear that the functions of various materials depend not only on the properties of the material itself, but also on the nanostructure inside the material.

Nanotechnology has a significant impact on material science, not only in observing miniscule efficacy and elucidating scientific phenomena in the nanoscale world. Accordingly, it is necessary to rethink material science while making use of knowledge of nanotechnology. In particular, the creation of functional structures through self-assembling processes such as supramolecular chemistry and the advances in nanotechnology that bring about an understanding of nanostructures are important elements for the development of functional materials. Recently, a new concept of nanoarchitectonics has been proposed as a unifying concept between nanotechnology and other scientific disciplines such as supramolecular chemistry. Just as Richard Feynman proposed nanotechnology in the 20th century, nanoarchitectonics was proposed by Masakazu Aono in the early 21st century. As a post-nanotechnology concept, nanoarchitectonics creates functional materials and systems through bottom-up processes and nanotechnology insights. Nanoarchitectonics is a methodology to architect functional material systems using nanoscale units such as atoms, molecules, and nanomaterials. The concept of nanoarchitectonics serves as a unifying concept that integrates nanotechnology with other research fields as post-nanotechnology.

Nanoarchitectonics is a highly universal concept. Because of its conceptual versatility, nanoarchitectonics can be applied to a wide range of research fields, including materials synthesis, structure formation, sensing, catalysis, environmental remediation, energy production and storage, device fabrication, and bio/biotherapy. Even without explicitly calling it nanoarchitectonics, the structure fabrication from molecules to materials that has been done so far can be considered part of the nanoarchitectonics process. Re-imagining these materials sciences with the new concept of nanoarchitectonics will promote a comprehensive understanding of related fields and lead to the discovery of new insights among interdisciplinary research fields. The purpose of this focus issue, titled as advancements of functional materials with nanoarchitectonics as post-nanotechnology concept in materials science, is to illustrate the importance of nanoarchitectonics as a post-nanotechnology concept by presenting outstanding examples from the progress of such research. Now is the time for a fundamental paradigm shift from nanotechnology to nanoarchitectonics.

In this focus issue, collected ten papers (one regular article and nine papers) exhibit a wide range of possibilities of nanoarchitectonics methodology both in basic science and application technologies. As fundamental materials nanoarchitectonics, Yokoi, Shimabukuro, and Kawashita discuss synthesis and characterization of octacalcium phosphate materials together with their physicochemical and biomaterial applications. For structure-target nanoarchitectonics, Grasset, Berling, Icon, and co-workers propose a novel strategy for direct writing laser patterning of photoluminescent ZnO materials in the deep-UV range. Investigation by different spectroscopic approaches revealed photocrosslinking-based mechanisms. Important roles of metal nitride-based nanostructures are focused in a review article by Singh and co-workers. In addition to synthetic strategies for metal nitride nanomaterials, electrochemical and photocatalytic application for hydrogen production are discussed. Park, Kim, and co-workers describes possibilities of carbon-based frameworks and their composites in different dimensionalities in lithium metal storage. Nanoarchitectonics and related materials designs of porous structures would be important keys for better performances. Zhang, Ye, and co-workers focus on hetero-interface nanoarchitectonics in design and fabrication of photocatalytic and electrocatalytic materials for water splitting applications. The hetero-interfaces nanoarchitectonics is often advantageous for promoted properties such as efficient charge separation, lower reaction energy barriers, faster transfer processes, and higher conductivity. A review article by Yamaguchi and Ogawa discusses photoinduced movements of various substances with a wide range of sizes from molecules, polymers to microscopic particles.

Bio-related applications with nanoarchitectonics concept are also included. A review article by Jia and coworkers discusses the fate regulation of mesenchymal stem cells by biomaterials nanoarchitectonics. Integration of stem cells and biological substances upon materials nanoarchitectonics have high potentials for tissue engineering and regenerative medicine. Effective roles of materials nanoarchitectonics in biological science and technology is also indicated by Leong and coworkers. As representative nanomaterials, various two-dimensional materials including fullerene nanosheets, biomimetic nanosheets, two-dimensional assemblies of fullerene nanowhiskers are exemplified together with their bio-interactive function. Furthermore, device applications can be targets of nanoarchitectonics approaches. Gimzewski, Stieg, and co-workers propose highly advanced utilization of nanoarchitectonics concept in computing systems. Their materials-nanoarchitected neuromorphic computing is based on polyaniline-integrated atomic switches. Park and co-workers discuss utilization of interactive structural color expression upon nanoarchitectonics block copolymers. Stimuli-interactive structural color displays can be nanoarchitectonically programmable with one-dimensional block copolymer photonic crystals.

The above-mentioned examples demonstrate a wide range of applicability of the nanoarchitectonics concept. In addition to the papers in this focus issue, the number of recent publications advocating nanoarchitectonics has increased dramatically. A review of these papers shows that the concept of nanoarchitectonics is widely applied from basic academic fields to applied fields. Since materials are composed of atoms and molecules, nanoarchitectonics is considered to be a universal unified concept that can be applied to all materials. Therefore, it can be likened to the theory of everything in physics, and can be called a method for everything in material science.

